# *A multi-day and high-quality EEG dataset for motor imagery* brain-computer interface

**DOI:** 10.1038/s41597-025-04826-y

**Published:** 2025-03-23

**Authors:** Banghua Yang, Fenqi Rong, Yunlong Xie, Du Li, Jiayang Zhang, Fu Li, Guangming Shi, Xiaorong Gao

**Affiliations:** 1 School of Mechatronic Engineering and Automation, Research Center of Brain-Computer Engineering, Shanghai University, Shanghai, China; 2 Engineering Research Center of Traditional Chinese Medicine Intelligent Rehabilitation, Ministry of Education, Shanghai, China; 3 Medical School, Tianjin University, Tianjin, China; 4 School of Artificial Intelligence, Xidian University, Xi’an, China; 5 Department of Biomedical Engineering, School of Medicine, Tsinghua University, Beijing, China

**Keywords:** Brain-machine interface, Neuroscience

## Abstract

A key challenge in developing a robust electroencephalography (EEG)-based brain-computer interface (BCI) is obtaining reliable classification performance across multiple days. In particular, EEG-based motor imagery (MI) BCI faces large variability and low signal-to-noise ratio. To address these issues, collecting a large and reliable dataset is critical for learning of cross-session and cross-subject patterns while mitigating EEG signals inherent instability. In this study, we obtained a comprehensive MI dataset from the 2019 World Robot Conference Contest-BCI Robot Contest. We collected EEG data from 62 healthy participants across three recording sessions. This experiment includes two paradigms: (1) two-class tasks: left and right hand-grasping, (2) three-class tasks: left and right hand-grasping, and foot-hooking. The dataset comprises raw data, and preprocessed data. For the two-class data, an average classification accuracy of 85.32% was achieved using EEGNet, while the three-class data achieved an accuracy of 76.90% using deepConvNet. Different researchers can reuse the dataset according to their needs. We hope that this dataset will significantly advance MI-BCI research, particularly in addressing cross-session and cross-subject challenges.

## Background & Summary

Brain-computer interface (BCI) represents a revolutionary technology, enabling users to control external devices and software applications by decoding neural activity, bypassing the need for muscular involvement^1,2^. These neural activities are captured and analyzed using non-invasive methods such as electroencephalography (EEG)^1^, functional magnetic resonance imaging (fMRI)^3^, and functional near-infrared spectroscopy (fNIRS)^4^. Among these techniques, EEG is particularly favoured in BCI systems due to its lower risk, cost-effectiveness, and parcticality^5,6^. BCI systems typically utilize paradigms such as steady-state visual evoked potential^7^, event-related potential^8^, and motor imagery (MI)^9^. Notably, MI is distinct in that it reflects the subject’s voluntary movement awareness without any actual physical motion^10^. Recent advancements have underscored the potential of BCI, particularly MI-based BCI (MI-BCI), in post-stroke motor rehabilitation, highlighting its significant therapeutic benefits^1,11–13^.

To promote technological innovation in EEG-based MI-BCI and neuroscience, enhance research transparency and scientific rigor, and foster interdisciplinary collaboration, many MI EEG datasets have been made publicly available. The BCI IV-2b^14^ and OpenBMI^15^ datasets are two representative examples, each focusing on two-class MI tasks. These datasets have played a key role in accelerating the validation and enhancement of EEG classification models. The BCI IV-2b dataset contains EEG data from 9 subjects, recorded using three electrodes (C3, C4, Cz). The OpenBMI dataset includes MI EEG data from 54 healthy subjects across three recording sessions, yet the average decoding accuracy across all subjects and sessions is only 74.7% when using the state-of-the-art algorithm^16^. Currently, the only widely used multi-task MI dataset for algorithm research is the BCI IV-2a dataset, which includes left-right hand, foot and tongue MI tasks, and was collected in 2008^17^. Like the 2b dataset, this 2a dataset also involves only 9 subjects and includes recordings from 22 electrodes.

Most existing datasets face challenges related to limited data diversity and suboptimal data quality. Common issues include a small number of subjects, a low channel count, limited recording sessions, a restricted range of tasks, and subpar classification accuracy. These shortcomings, particularly the small sample sizes, significantly hinder the development and validation of advanced algorithms. Moreover, inadequate sample sizes or single-task designs pose barriers to technological progress and iterative advancements in the MI-BCI field. With the application of deep learning algorithms in the MI-BCI field^18^, there is an increasing demand for large scale, high quality EEG datasets. In this paper, we introduce a comprehensive MI-BCI dataset comprising data from 62 subjects across three recording sessions. This dataset includes two paradigms: the first involves upper limb movements with left and right hand-grasping, with 51 subjects participating; the second adds a foot-hooking task, involving 11 subjects. For clarity, we refer to the two-task paradigm dataset as 2 C and the three-task paradigm dataset as 3 C. The average decoding accuracies across all subjects and sessions for the 2 C and 3 C data, respectively, using state-of-the-art algorithms are 85.3% and 76.9%. The public dataset consists of raw data, processed data and the associated code. These have been made publicly available on Figshare to ensure wider accessibility, with access provided through a dedicated link^19^ (10.25452/figshare.plus.22671172).

Based on this dataset, we compare the differences in EEG activation patterns between different tasks from a temporal-spectral-spatial perspective, and utilize both existing baselines and state-of-the-art methods to perform decoding tasks, demonstrating that the quality of this dataset surpasses that of currently available public datasets. Also, we observed that the MI ability of different subjects improved progressively after multiple MI sessions. More deeply, due to the well-distributed performance of the 2 C dataset, future development of this dataset could also focus on BCI illiteracy, exploring the differences between high performers and low performers.

We hope that this dataset will serve as a significant and robust resource for advancing of MI-BCI systems, owing to its high quality and large scale. Its versatility makes it an invaluable tool for both traditional and deep learning approaches in MI-BCI research. This includes applications in subject-specific^20^, subject-independent^21^, and session-to-session transfer studies^22^, as well as in the development of transfer models.

## Methods

### The 2019 world robot conference contest-BCI robot contest MI

The “World Robot Conference Contest-BCI Robot Contest MI (WBCIC-MI)” serves as a platform to promote advancements in BCI technology and foster interdisciplinary collaboration. The two datasets presented in this study were collected during the 2019 edition of WBCIC-MI, where participants engaged in standardized MI tasks under controlled conditions. The contest provided a unique environment for collecting high-quality, large-scale EEG data, contributing significantly to the development of this comprehensive dataset.

### Participants and environment

In this experiment, 62 healthy, right-handed participants (aged 17–30, including 18 females), all naive BCI users, were recruited. Among them, 51 subjects participated in the two-class MI experiment, and 11 in the three-class MI experiment. None had a history of neurophysiological, psychiatric, or musculoskeletal disorders that might have influenced the results. Prior to the experiment, all subjects are thoroughly informed about the procedure, purpose, requirements (such as maintaining health, adequate sleep, abstaining from alcohol), and MI techniques. After ensuring their understanding, written informed consent is obtained. The study receives approval from the Tsinghua University Medical Ethics Committee (approval number: 20190002) and adhered to the Declaration of Helsinki principles.

The participants consist of university students. The recruitment information includes details about the experiment, its purpose, instructions, and potential risks involved (mainly physical fatigue). This targeted recruitment aims to minimize age variability and offers an educational opportunity in BCI technology. Participant privacy is strictly protected, with voluntary participation assured, and the commitment that data from any withdrawing participants would not be used. No participants are harmed, but provisions for compensation and treatment were established in case of harm.

The participants consist of university students. The recruitment information includes details about the experiment, its purpose, instructions, and potential risks involved (mainly physical fatigue). This targeted recruitment aims to minimize age variability and offered an educational opportunity in BCI technology. Participant privacy is strictly protected, with voluntary participation assured, and the commitment that data from any withdrawing participants would not be used. No participants are harmed, but provisions for compensation and treatment were established in case of harm.

### Experimental paradigm

**Fig. 1:**
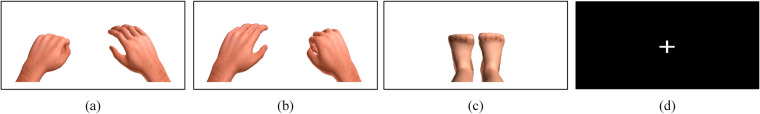
The representations of visual cues according to each task. (**a**) Left-hand grasping. (**b**) Right-hand grasping. (**c**) Foot-hooking. (**d**) Break.

The two experiment paradigms are designed to quantitatively acquire data related to different limb MI tasks. The experimental tasks comprise three tasks: left hand-grasping, right hand-grasping and foot-hooking. In particular, foot-hooking refers to keeping the heel of the foot stationary while slowly lifting the toe, creating a 45-degree angle with the ground. At the beginning of each experiment, visual and auditory instructions appeared simultaneously. The visual cues of MI tasks are provided on the monitor by displaying a brief video on a white background, while the resting cue is displayed as a white cross sign on a black background, as shown in Fig. 1.

**Fig. 2:**
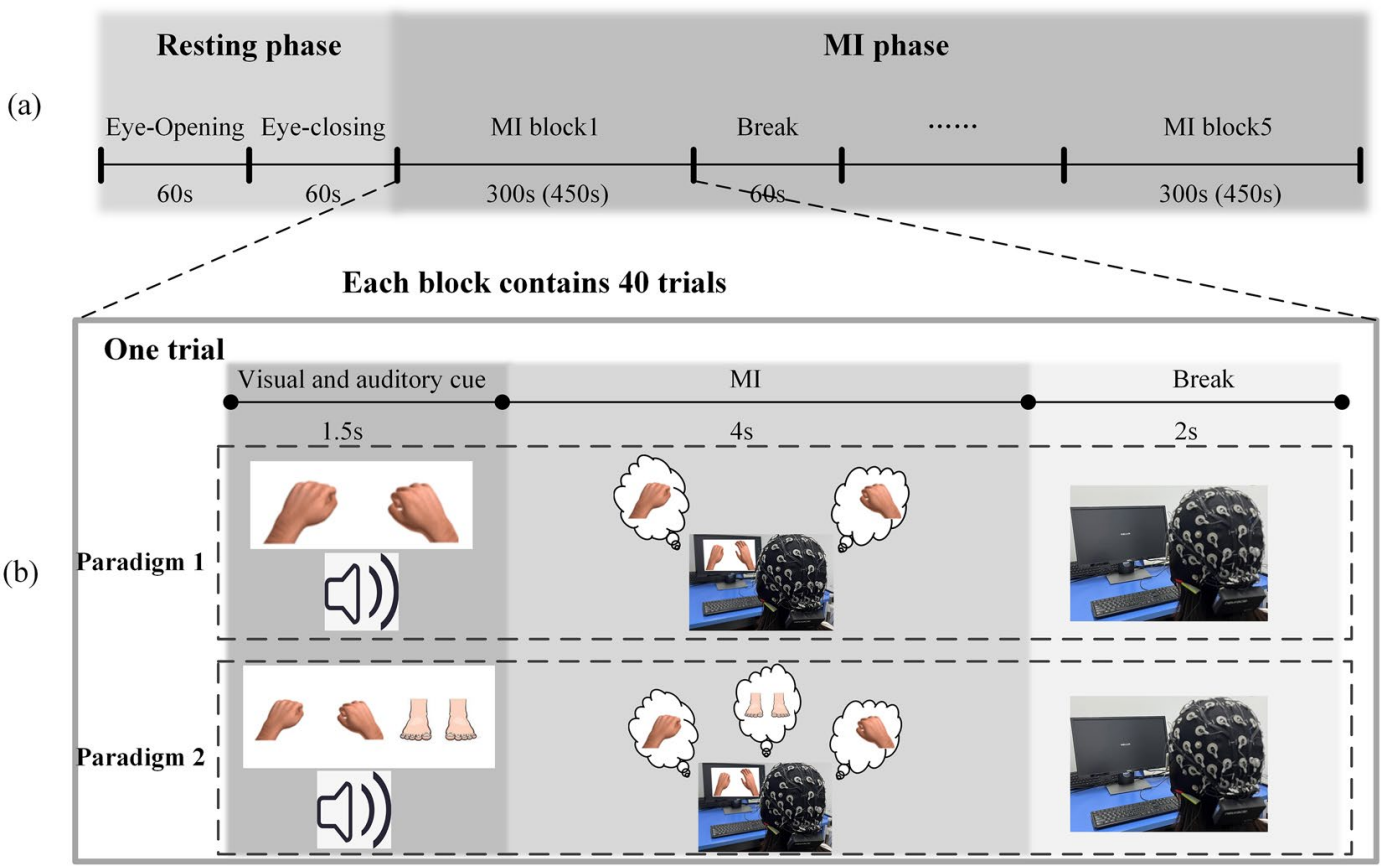
Experimental Paradigm of MI. (**a**) The experimental paradigm includes a resting phase and a MI phase, with the MI phase comprising five MI blocks. (**b**) Experimental paradigm in a single trial.

Each subject does three recording sessions with the same MI paradigm on different days. Our experiments comprised multiple recording sessions (three days) to consider inter-session and inter-participant variabilities. Each recording session last about 35–48 minutes, which includes eye-opening (60 s), eye-closing (60 s) and five MI blocks as indicated in Fig. 2(a). We provide a flexible break period between the two MI blocks owing this experiment required subjects to remain focused for a long time. After the 60 s of break, the participants can choose to start the next MI block or continue to break according to their conditions.

After the tasks of eye-opening and eye-closing, the MI task is started. In the 2 C dataset, there are 40 trials in each block with balanced left-right hand MI tasks (3 C dataset: 60 trials, each block with balanced left-right hand and foot-hooking MI tasks). The duration of each trial is 7.5 s, which is shown in Fig. 2(b). The first 1.5 seconds of a single trial involve brief visual and auditory cues. After the brief cues, participants perform the corresponding MI task during the MI period based on these cues, lasting for 4 seconds. During the MI period, participants are required to mentally repeat the imagined tasks 2–4 times based on the given cues. The subjects stop the MI task, when the monitor displays a white cross sign on a black background. The break lasts for 2 seconds.

It is important to note that there are no unnecessary visual and auditory stimuli throughout the entire experimental process. As shown in Fig. 2, each MI block consists of 40 trials. Therefore, there are 200 trials pear recording session of 2 C dataset, with 100 trials for each MI task (left-right hand grasping). And there are 300 trials pear recording session of 3 C dataset, with 100 trials for each MI task.

### Data collection

**Fig. 3:**
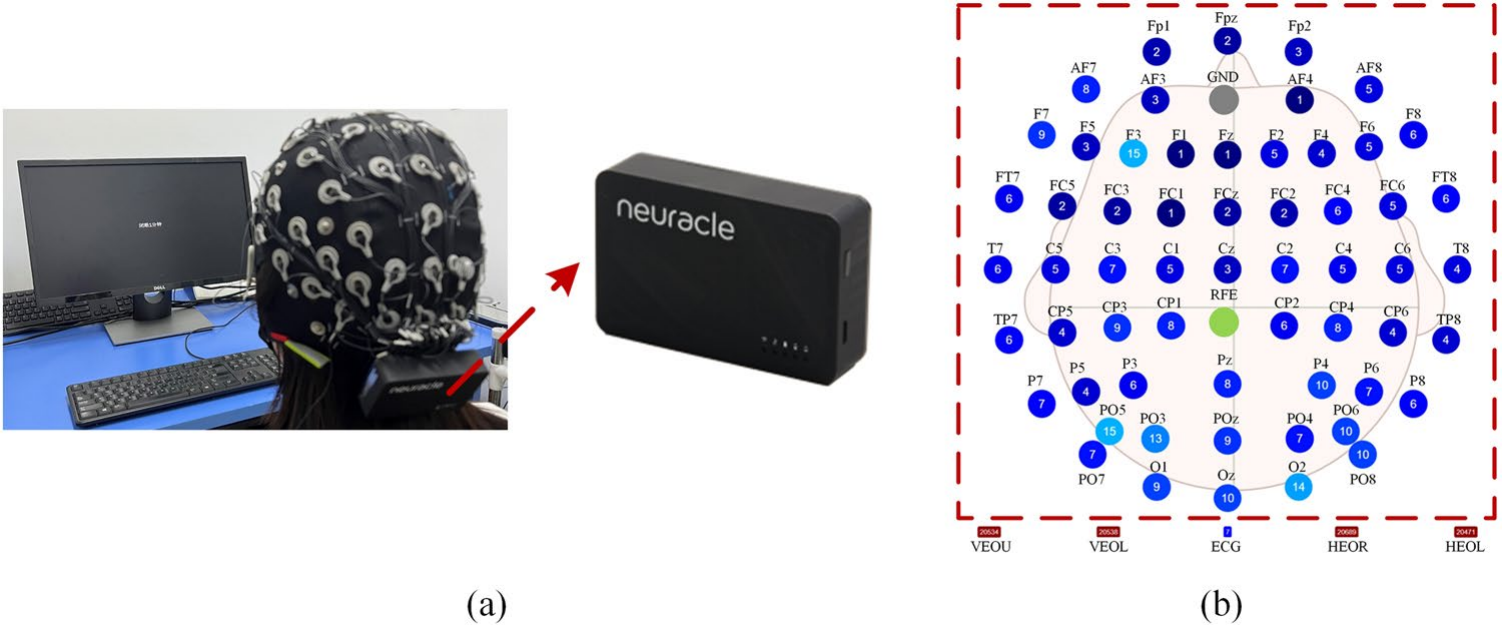
The data collection equipment used in this experiment. (a) The EEG cap and amplifier used in the experiment. (**b**) The electrode positions on the EEG cap and impedance values for one participant before the experiment, unit: kΩ. Where, the gray dot represents ground electrodes, while the green one represents reference electrodes. VEOU: Vertical electrooculography upper. VEOL: Vertical electrooculography lower. HEOR: Horizontal electrooculography right. HEOL: Horizontal electrooculography left.

An EEG cap with 64 channels is placed on the head of each subject, which is a new generation of wireless EEG equipment independently developed by Neuracle (http://www.neuracle.cn/productinfo/148706.html)^23–25^, as shown in Fig. 3(a). The system is characterized by good portability, signal stability, and effective shielding. More information about the amplifier and the system is presented in Table 1. The electrode positions of the EEG cap are arranged according to the international 10–20 system. Of the 64 channels, 1–59 record EEG signals, and channels 60–64 record electrocardiogram (ECG) (60) and electrooculogram (EOG) (61–64) signals, respectively.

**Table 1:** Information about the amplifier and the system used in this study.

Technical specifications	
Sampling Rate:	Maximum single-channel sampling frequency of 16 kHz
Common Mode Rejection Ratio:	120 dB
ADC Resolution:	24bits
Bandwidth:	DC amplification preserving the full bandwidth signal, DC-4kHz under 16 kHz sampling
Input Noise:	<0.4uVrms (0.3~70 Hz)
Input Range:	+/−375 mV
Data Transmission:	WIFI transmission, supports 2.4 GHz/5 GHz dual-band transmission
Data Sync Accuracy/Time Accuracy:	<1 ms
Power Supply:	Lithium battery
EEG Cap Waterproof Rating:	IPX8, can be quickly cleaned and dried with specialized equipment
Weight:	84 g
Pose Detection:	Pose Detection
Anti-interference Capability:	Excellent electromagnetic shielding, capable of operating in various complex environments

As shown in Fig. 3(b), the electrode positions for EEG collections are indicated within the red box, while the rest pertain to ECG and EOG. The ECG and EOG electrodes are not used during these two experiments. During the experiment, to obtain high-quality signals, the impedance for all channels is kept as low as possible, preferably below 5 kΩ, and the sampling frequency is set to 1000 Hz. The impedance values for all channels of one participant before the experiment are presented in Fig. 3(b), the numbers inside the circles represent the impedance values for the corresponding channels, measured in kΩ.

The experimental paradigms outlined in this study are implemented using E-Prime^26^, a comprehensive software suite designed for creating and conducting psychological and neuroscientific experiments. E-Prime offers a user-friendly graphical interface, facilitating the entire process from the generation of experiments to the collection of data with millisecond precision. It also enables preliminary data analysis. The software’s versatility allows for the presentation of various stimuli, including text, images, and sounds. Additionally, E-Prime provides detailed timing information and logs event specifics, which are crucial for the accuracy and reliability of experimental data in neuroscience research.

### EEG data pre-processing

The aforementioned system provides continuous raw EEG data. The raw data contains irrelevant channels and a high sampling frequency (1000 Hz). Therefore, pre-processing methods are required to obtain cleaner EEG data. In this paper, the data pre-processing is conducted using the EEGLAB (v2023.0) toolbox in the MATLAB (R2021b) environment. The specific pre-processing methods are as follows:

- Select data. Even though the ECG and EOG channels did not record any data during the MI experiments, these five channels were still included in the final EEG data. Hence, the first step of pre-processing is to remove the irrelevant channels, such as ECG (60) and EOG (61–64) (In this case, the number of data channels is 59).
- Re-reference. To avoid information loss, further to ensure consistency and comparability of results, the data from all participants underwent re-referencing. For re-reference of EEG data in this paper, Pz is used as the reference electrode (In this case, the number of data channels is 58 and after testing, this re-reference method is the best method).
- Filtering. To eliminate power line interference and high-frequency noise during the experiments, the filtering process is applied to the data. In this study, we used finite impulse response (FIR) in EEGLAB to perform band pass filtering (0.5–40 Hz) and filtering of 50 Hz. When the cutoff frequency is less than 2 Hz, the cutoff frequency is set to twice its value. This can help better eliminate low-frequency noise or baseline drift, ensuring that the signal retains the desired high-frequency components.
- Extract epochs. What we’re interested in is the data segment that makes the stimulus. The four seconds after the visual and auditory cue ends are the period we are focusing on. The MI section shown in Fig. 2(b) represents the valid epochs we extracted. At this point, the EEG data of each subject is divided into 200 trials on the 2 C dataset (3 C dataset: 300 trials) based on event markers.
- Remove epoch baseline. EEG signals contain baseline interference signals (low frequency noise), which can adversely affect signal analysis. Removing the epoch baseline is also a key step in pre-processing.
- Change sampling rate. In order to reduce the amount of data and improve the calculation speed, the data are down-sampled to 250 Hz.

## Data Records

**Fig. 4:**
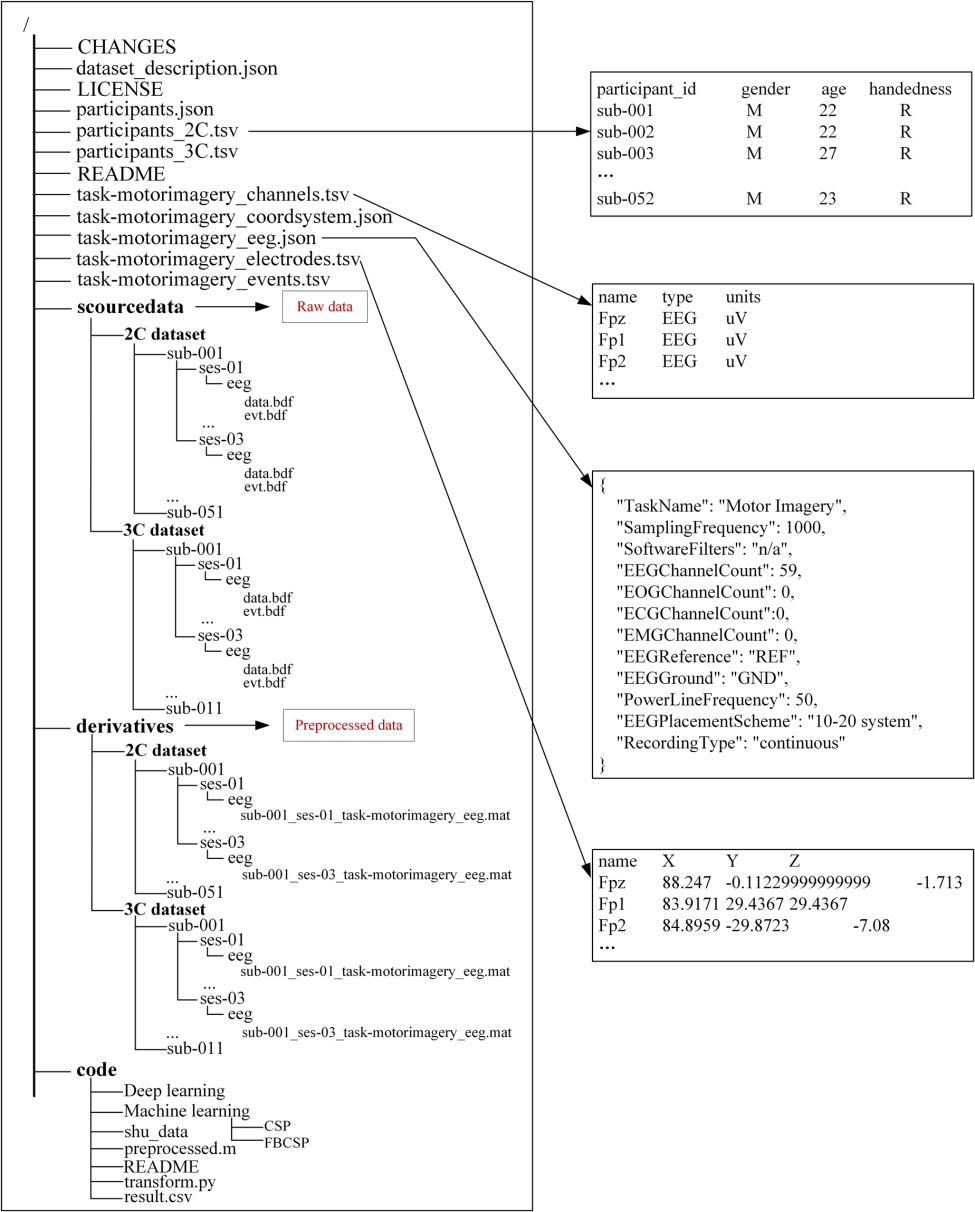
Directory tree for the repository with previews of EEG files.

The dataset is available at Figshare^19^. The source files and meta-data files in this dataset were organized according to EEG-BIDS^27^. The directory tree for this repository and some previews for data are shown in Fig. 4.

On the whole, the repository’s data consists of two parts: (1) Raw data is stored in the home folder – ‘scourcedata’; (2) Processed data store in the derivatives folder – ‘.mat’ files. Within these directories, the subdirectory corresponding to each subject is named “sub-xxx”, where xxx represents the serial number of the subject.

### Raw data

The acquired raw data by Neuracle for a single task session are saved as ‘.bdf’ and organized according to the following naming rules:

sub-xxx\ses-yy\eeg\data.bdf,

sub-xxx\ses-yy\eeg\evt.bdf,

where, xxx represents for the subject number (2 C dataset: 001, 002, …, 051; 3 C dataset: 001, 002, …, 011), yy is the recording session number (01, 02, 03). There were two ‘. bdf’ files in each folder, which are the data file (data.bdf) and the trigger file (evt.bdf). It is important to note that naming the raw data as “data” and “evt” ensures that the data can be properly opened in EEGLAB.

### Processed data

The processed data are obtained through the EEGLAB (v2023.0) toolbox based on MATLAB (R2021b). After pre-processing steps, the data for each recording session is stored as one file. The naming rule for each file is as follows:

sub-xxx_ses-yy_task-motorimagery_eeg.mat,

Each ‘.mat’ file contains two variables:

data: 200 or 300 trials of MI data (2 C dataset: 200 trials, 3 C dataset: 300 trials), there are 100 trials for each class of MI task. Its dimension was [58 × 1000 × 200] or [58 × 1000 × 300], which is channel numbers × time samples × trial numbers.

labels: It contains the task triggers (2 C dataset: ‘1’ and ‘2’, 3 C dataset: ‘1’, ‘2’and ‘3’).

## Technical Validation

We evaluate the dataset through time-frequency-space domain visualization and commonly used classification algorithms in the MI-BCI field.

During MI, the cerebral cortex undergoes significant changes in specific rhythm signals, notably the mu-rhythm (8–12 Hz) and beta-rhythm (13–30 Hz)^28^. These signal alterations play a crucial role in understanding the neural mechanisms underlying MI. We focus on the Event-Related Desynchronization/Synchronization (ERD/ERS) features, which are essential in MI tasks. ERD/ERS features differ in their spatial distribution patterns across the brain depending on the limb involved in the MI task. These patterns correspond to the distribution of the cortical motor areas associated with the active limbs^29^.

In addition to visual analysis, evaluating the quality of MI data using advanced algorithms is an effective approach. The performance of classification algorithms can serve as an indirect measure of the data’s quality^30^. The overall classification performance can reveal the usability and the quality of the dataset as a whole.

### Temporal domain

Recent studies have increasingly validated the utility of ERD/ERS as effective analytical methods for studying brain functional signals. In our research, we specifically focus on the ERD/ERS of the mu-rhythm (8–12 Hz), which is closely associated with limb sensory-motor activities. This rhythm can be modulated by sensory stimulation and motor activities. The process for calculating ERD/ERS in our study was methodically structured as follows: (1) The 8–12 Hz band pass filtering is performed on the EEG signal; (2) each sample point of the filtered EEG signal is squared; (3) The squared EEG signals corresponding to the same MI task are superimposed and averaged; (4) The average curve is smoothed using a sliding time window. (5) The ERD/ERS metric was obtained using the formula:

1 $${ERD}/{ERS}=\frac{A-R}{R}\times 100 \% $$

here, $A$ represents the power spectral density (PSD) of the EEG signal in a specific frequency band during MI, while $R$ represents the PSD of the same frequency band of the EEG signal during the reference period (resting state). $A$ and $R$ are both vectors.

### Spectral domain

The MI-EEG also showed obvious performance in the spectral domain. The event-related spectral perturbation (ERSP) is used to reflect the changes of energy during MI tasks^31^. ERSP is a method used in EEG analysis to examine the changes in the spectral power of brain oscillations in response to specific events or tasks. It provides a time-frequency representation of the EEG signal and allows researchers to investigate the dynamic modulation of neural activity during different MI tasks. ERSP involves calculating the spectral power at different frequencies and time points relative to a baseline period. The baseline period is typically a pre-task interval where the participant is at rest or engaged in a neutral state. By comparing the spectral power during the task or event to the baseline, it can determine the event-related changes in power at specific frequency bands and time intervals. The calculation process for ERSP involved the following steps: (1) Apply the short-time fourier transform (STFT) to the time-frequency decomposition of preprocessing data; (2) Calculate the energy values for each time point and frequency bin in the time-frequency representation; (3) Use the time before the start of the MI tasks as the baseline period; (4) Compute the average energy values during the event of interest and the baseline period. The formula for calculating ERSP is as follows,

2 $${ERSP}\left(f,t\right)=\frac{1}{n}\mathop{\sum }\limits_{k-1}^{1}{\left|{F}_{k}\left(f,t\right)\right|}^{2}$$

here, ${F}_{k}(f,t)$ is the spectral estimate of trial $k$ at frequency $f$ and time $t$.

### Spatial domain

The common spatial pattern (CSP) is a widely used method in the analysis of EEG and other biological signals. It is particularly effective in extracting spatial patterns that show maximum differences between distinct brain states. After preprocessing the EEG signals, the CSP algorithm is applied to extract spatial features by computing a set of spatial filters. In the CSP algorithm, a set of spatial filters is computed based on the covariance matrices of EEG signals for different conditions or tasks. The goal of CSP is to find a set of spatial filters that maximize the variance of the signals from one class and minimize the variance of the signals from the other class. This is done by solving the following optimization problem:

3 $${\arg }\,{\max }\sum _{i,j}\frac{{var}\left({X}_{i}W\right)}{{var}({X}_{j}W)}$$

where, ${X}_{i}$ represents the EEG signal matrices for different class, $W$ is the matrix of spatial filters. *var*(*X*_*i*_ *W*) and *var*(*X*_*j*_ *W*) are the variance of the projected signals for the respective classes.

The $W$ resulting spatial filters enhance or suppress specific spatial patterns that are most useful for discriminating between classes. These spatial components are then used as features for classification, which helps to improve the performance of the BCI system by focusing on the brain areas most relevant to the MI tasks. This method of spatial feature extraction enhances the discriminability of the brain states, making it a powerful tool for MI-BCI applications.

### Visualization results

**Fig. 5:**
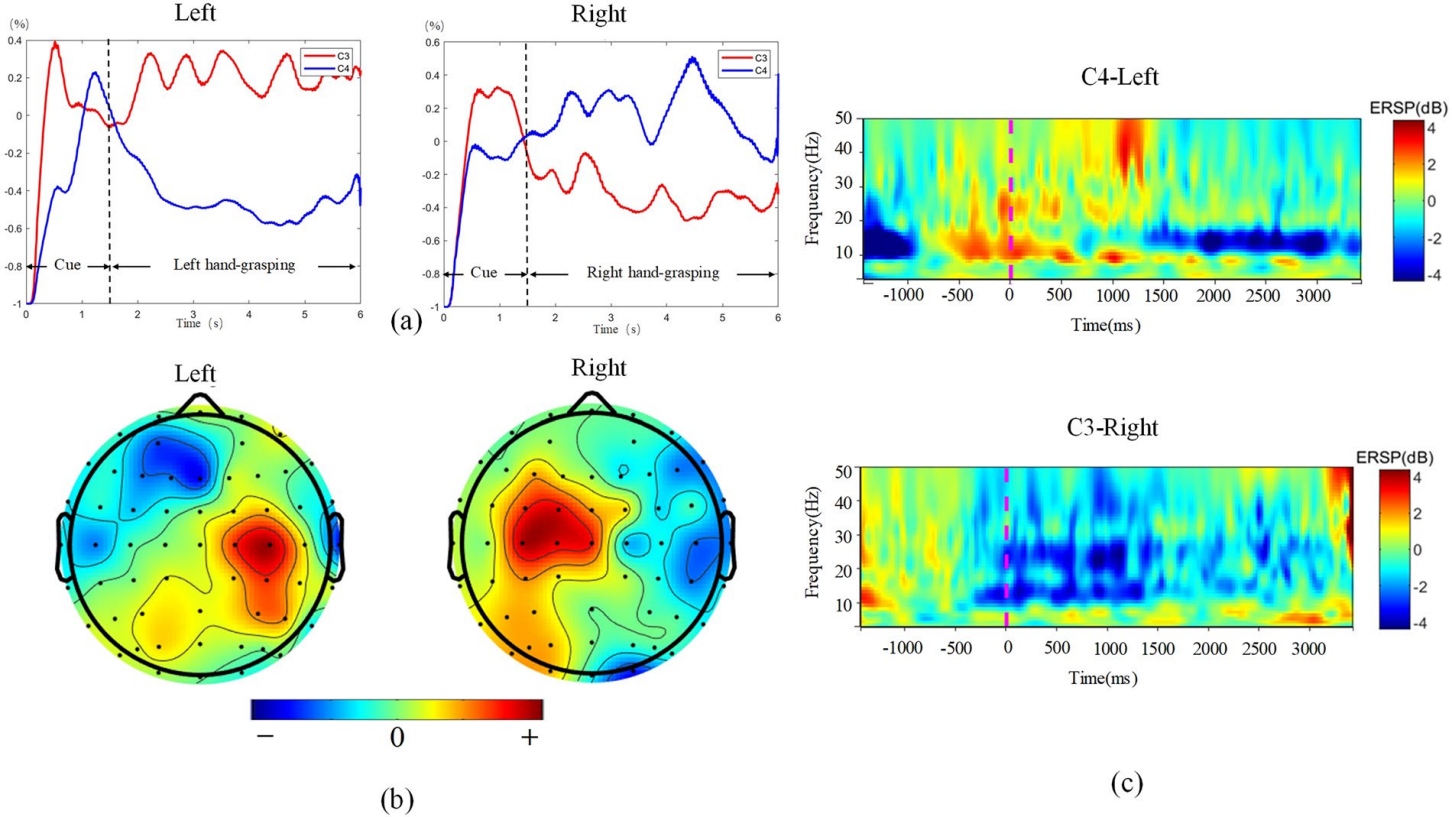
The visualization results of the 2 C dataset. (**a**) ERD/ERS. (**b**) Topography map, the colorbar represents the level of activation, with “+” indicating activation and “−” indicating deactivation. (**c**) ERSP.

**Fig. 6:**
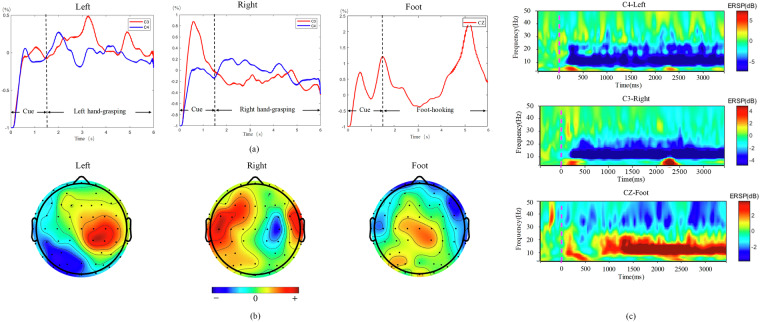
The visualization results of the 3 C dataset. (**a**) ERD/ERS. (**b**) Topography map, the colorbar represents the level of activation, with “+” indicating activation and “−” indicating deactivation. (**c**) ERSP.

Figures 5, 6 display the visualization of temporal-spectral-spatial domains. Figure 5 illustrates the visualization results of the 2 C dataset. Figure 5(a) depicts the ERD/ERS phenomenon in the time domain. The result is the average across all participants, with the baseline obtained during the 1-second period preceding the onset of the MI task. From Fig. 5(a), it can be observed that the 2 C dataset exhibits a distinct ERD/ERS phenomenon. When imagining left hand-grasping, there is an increase in amplitude at electrode C3 and a decrease at C4, while imagining right hand-grasping shows the opposite pattern.

The ERD phenomenon is more pronounced than ERS, as depicted in Fig. 5(c), which shows the ERSP for the C4 channel during the left hand-grasping task and the C3 channel during the right hand-grasping. The ERSP in Fig. 5(c) is consistent with the ERD/ERS observed in 5(a). The ERSP plot illustrates spectral variability according to the time epoch in the C3 and C4 channels, which reflect sensorimotor activation and deactivation. The reduction in energy during left and right hand-grasping occurs within the 8–30 Hz range. Additionally, it is observed that the reaction time for imagining the right hand is faster than the left hand, which may be related to the fact that all participants are right-handed.

Further analysis is performed by plotting selected spatial patterns of the CSP feature pairs (mu-rhythm) from all subjects in Fig. 5(b). The left (right) hand MI resulted in the activation of the region around the right (left) motor cortex. Figure 5(b) validates this statement. At the same time, it can be observed from Fig. 5(b) that the MI task not only activates the motor area but also elicits activation in the visual area.

Figure 6 presents the visualization results of the 3 C dataset. Unlike the 2 C dataset, the 3 C dataset includes a foot-hooking task. The analysis results for the ERD/ERS, ERSP, and topography maps for left and right hand tasks are consistent with those of the 2 C dataset. It is noteworthy to mention the dual-foot task. From Fig. 6(a), it can be observed that the dual-foot task exhibits an ERS phenomenon in the Cz channel. Figure 6(c) reflects the reduction in energy in the 8–30 Hz range for the foot-hooking task in the Cz channel. During the imagination of dual-foot movement, the visual area is also activated, as shown in Fig. 6(b).

### Classification performance

In assessing the classification performance of EEG signals, we employ traditional machine learning and deep learning methods. To uphold the standards of fairness and accuracy in our evaluation, a 10-fold cross-validation (CV) method is rigorously implemented^32^. The 10-fold CV facilitates a more comprehensive assessment of both the 2 C dataset and the 3 C dataset. This approach not only provides insights into the strengths and limitations of each dataset but also guarantees that our evaluation is robust and representative of diverse data scenarios.

In our study, traditional machine learning algorithms are employed, specifically the CSP^31^ and filter bank common spatial pattern (FBCSP)^33^ for feature extraction, paired with support vector machine (SVM)^33^ for classification. These methods are widely recognized as the benchmark algorithms in the field of MI-BCI^34^.

- CSP is adept at extracting spatial distribution components from multi-channel EEG data. Its core principle involves using matrix diagonalization to identify optimal spatial filters. This process maximizes the variance between two-class of signals, thereby yielding highly discriminative feature vectors. When task complexity increases (e.g., in multi-class classification), the performance of the CSP tends to decrease.
- FBCSP is proposed on the basis of CSP for processing MI-EEG data. This approach computes spatial filters in a supervised manner, focusing on band power features within the EEG signals for improved classification. FBCSP involves subdividing the input signal into multiple frequency bands using band-pass filters, with SVM serving as the classifier. The FBCSP typically performs better when handling more complex tasks and multi-class problems.

In the evolving landscape of EEG-BCI research, several deep learning architectures have gained prominence in recent years^35^. Notably, deepConvNet^36^, EEGNet^37^ and FBCNet^16^ are three architectures that have been widely adopted within the EEG research. Their popularity is partly attributed to the availability of open-source code implementations, which has facilitated their widespread use and application in various EEG-BCI studies.

- The EEGNet is a specialized, compact convolutional neural network (CNN) architecture, explicitly designed for EEG-BCI. Its compact design allows for effective learning from relatively small datasets common in BCI research, while its convolutional layers are adept at capturing both temporal dynamics and spatial patterns inherent in EEG signals.
- The deepConvNet represents an advanced deep learning architecture, specifically designed to directly learn and interpret both temporal-domain and spectral-domain features of EEG signals. By integrating learning from both domains, the deepConvNet offers a comprehensive understanding of EEG data, enabling more nuanced and accurate EEG signal interpretation.
- The FBCNet introduces an innovative variance layer within its architecture, specifically engineered to aggregate temporal-domain information from EEG signals effectively. The variance layer operates by computing statistical measures across time, emphasizing changes and patterns in the EEG signal over specific intervals. This novel feature positions FBCNet as a potent tool in the domain of EEG-BCI research, enhancing the depth and precision of EEG data analysis.

In order to analyze the classification performance of dataset more effectively and reasonably, the three deep learning algorithms adopt the same training parameters. The chosen parameters for each algorithm included a batch size of 16, a learning rate of 0.001, a loss function of NLLLoss, and the Adam optimizer. The simultaneous validation of these deep learning methods on both datasets further provided a comprehensive view of their applicability and effectiveness in varied data scenarios.

To ensure uniformity in our evaluation, we employed the same training procedure for all deep learning algorithms. A two-stage training strategy was used^16^, where the training data was further split into training and validation sets. Specifically, during the training process, 80% of the data is used for training the model, 10% is used for validation to tune hyperparameters and monitor performance, and the remaining 10% is reserved for testing to assess the final model’s generalization capability. During the first stage, the model was exclusively trained on the training set, with its performance continuously monitored using the validation set accuracy. The training process was halted if the validation set accuracy did not improve for 200 consecutive epochs, and the network parameters corresponding to the highest validation set accuracy were restored. This approach ensured that the model did not overfit and retained the best-performing parameters. Subsequently, the second stage of training commenced using the restored model as the starting point. In this stage, the model was trained on a combined dataset comprising both the training set and the validation set (referred to as the training data). The second stage of training was terminated when the test set loss fell below the loss achieved during the first stage. This two-stage strategy allowed the model to further refine its parameters while maintaining robust generalization performance. The maximum number of training epochs was restricted to 1500 and 600 for training stages 1 and 2, respectively. We performed cross-validation to assess the performance of all algorithms.

Accuracy is indeed a very effective and intuitive evaluation metric, and it is the most commonly used one. The formula is as follows:

4 $${\rm{Accuracy}}\left( \% \right)=\frac{{TP}+{TN}}{{TP}+{TN}+{FP}+{FN}}\times 100 \% $$

Here, *TP* represents the true positive, *TN* represents the true negative, *FP* stands as the false positive, and *FN* stands as the false negative.

**Fig. 7:**
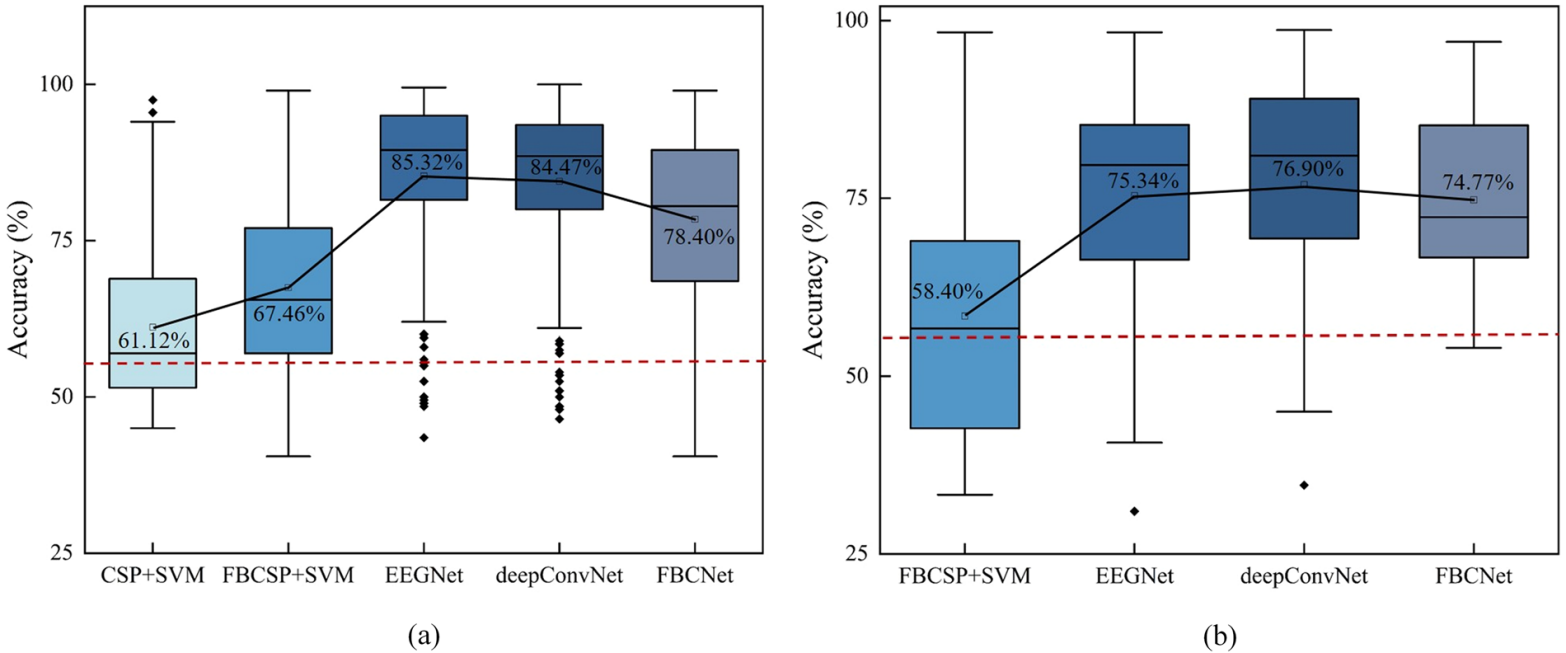
Classification accuracy of 2 C and 3 C dataset. The red dash-dotted line indicates chance level with p = 0.01^38^. (**a**) Classification accuracy of 2 C dataset. (**b**) Classification accuracy of the 3 C dataset.

Figure 7 shows the average classification accuracy of the two datasets under different benchmark algorithms, with the input data consisting of preprocessed 58-channel recordings. Figure 7(a) in our study illustrates the average classification accuracy across 153 independent recording sessions of the 2 C dataset, utilizing five different algorithms. In contrast, Fig. 7(b) depicts the average classification accuracy for 33 independent sessions of the 3 C dataset, analyzed using four algorithms. In the analysis conducted on the 2 C dataset, the classification accuracies achieved by each algorithm are notably distinct, with 61.12% (CSP + SVM), 67.46% (FBCSP + SVM), 85.32% (EEGNet), 84.47% (deepConvNet) and 78.40% (FBCNet). The classification accuracies achieved by each algorithm on 3 C dataset are 58.40% (FBCSP + SVM), 75.34% (EEGNet), 76.90% (deepConvNet) and 74.77% (FBCNet), respectively.

It can be seen that EEGNet exhibits superior classification performance on both datasets. It is important to note that each recording session comprised only 200 trials (2 C dataset) or 300 trials (3 C dataset), a relatively small dataset for deep learning algorithms. However, EEGNet’s performance underlines its effectiveness in handling limited training data, setting it apart as a robust choice for scenarios where data availability is constrained. These results emphasize the adaptability of EEGNet for efficiently processing smaller datasets, a common challenge in EEG-based BCI research.

Analysis of the classification accuracy across different recording sessions revealed a distinct trend related to participant experience with BCI systems. Table 2 shows the average classification accuracy across three sessions, obtained through EEGNet. From Table 2, it can be seen that in both datasets, the classification accuracy of session 1 is the lowest (2 C dataset: 81.77%, 3 C dataset: 71.91%), while that of session 3 is the highest (2 C dataset: 88.90%, 3 C dataset: 83.27%).

**Table 2:** The average classification accuracy across three recording sessions on the 2 C dataset and 3 C dataset.

Average accuracy			
	Session1	Session2	Session3
2 C dataset	81.77%	86.63%	88.90%
3 C dataset	71.91%	75.52%	83.27%

**Fig. 8:**
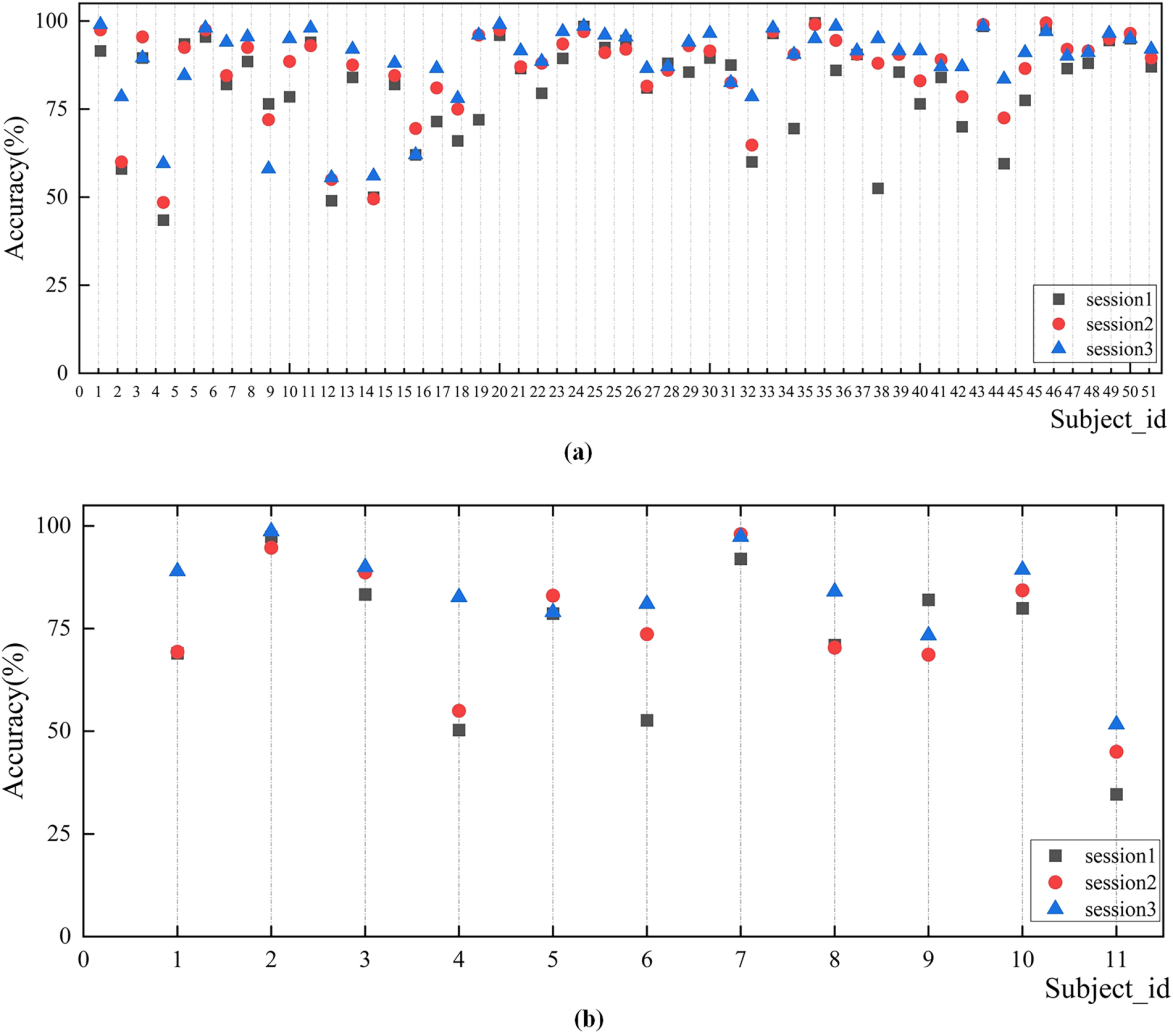
Scatter plot of classification accuracy for three recording sessions of the 2 C dataset and 3 C dataset. (**a**) Classification accuracy of 2 C dataset. (**b**) Classification accuracy of the 3 C dataset.

Furthermore, Fig. 8 displays the classification accuracy of all subjects across three recording sessions. As depicted in Fig. 8(a), the classification accuracy achieved for 51 subjects generally increased from the first to the third recording session. This pattern is indicative of the subjects’ growing familiarity and proficiency with MI tasks. Most subjects have higher accuracy in the third recording session, indicating that subjects had gained MI experience in the first two recording sessions (such as S1, S2, S4, S10, S19 and so on). There are a small number of subjects with high data classification performance for all three recording sessions (S3, S6, S20, S24, S26 *et al*.). This could reflect a natural aptitude. However, there are exceptions, such as Subjects S9 and S16, who showed poor performance in the third session, indicating variability in individual learning curves.

Similarly, the 3 C dataset, as illustrated in Fig. 8(b), exhibited comparable characteristics. These observations provide valuable insights for researchers utilizing this dataset, offering a unique opportunity to delve deeper into factors influencing learning and adaptation in BCI tasks.

## Discussion

To further assess the quality and applicability of the dataset, we compare its classification performance with two widely used EEG datasets: the BCI IV-2a dataset^17^ and the OpenBMI dataset^15^. These datasets were chosen for their similarity in experimental design and task paradigms. The most important dataset characteristics are summarized in Table 3.

**Table 3:** Comparative summary of selected dataset’s characteristics from the literature.

	Dataset	# of subjects	# of session	# of classes	# of channels
Existing	BCI IV-2a	9	2	4	22
	OpenBMI	54	2	2	20
This paper	2 C	51	3	2	58
	3 C	11	3	3	58

For this comparison, we employed the same preprocessing and classification algorithms across all datasets. The classification accuracies of the datasets were then evaluated using a consistent evaluation framework. Table 4 shows the average classification accuracies for the three datasets using the same algorithms. Our dataset achieved an average classification accuracy of 85.31% for the 2 C dataset and 76.90% for the 3 C dataset. In comparison, the BCI IV-2a dataset achieved 79.03% (four-class), while the OpenBMI dataset achieved 74.70% (two-class).

**Table 4:** The average decoding accuracies across all subjects and sessions by using data from selected datasets.

Dataset	EEGNet	deepConvNet	FBCNet
BCI IV-2a	73.13%	72.20%	79.03%
OpenBMI	70.89%	68.33%	74.70%
2 C	85.31%	84.47%	78.40%
3 C	75.34%	76.90%	74.77%

The comparison demonstrates that our dataset outperforms the BCI IV-2a and OpenBMI datasets in terms of overall evaluation, including the number of subjects, the number of recording sessions, the number of channels, and classification accuracy. This indicates that our dataset not only provides higher-quality data but also has greater potential for supporting the development of robust MI-BCI systems. The superior performance may be attributed to the larger number of subjects, the inclusion of multiple tasks, and the high-quality signal recording across three sessions.

The technical validation through comparison with existing datasets confirms the high quality and potential of our dataset for MI-BCI research. Future studies will further explore how this dataset can contribute to advancing classification algorithms and improving the robustness of MI-BCI systems.

## Usage Notes

This comprehensive MI-EEG dataset comprises two subsets: the 2 C dataset and the 3 C dataset. Users can readily download both the datasets and the accompanying code. The ‘code.rar’ file encompasses five benchmark algorithms along with classification results for all subjects and sessions. The ‘2 C dataset.rar’ and ‘3 C dataset.rar’ files contain both raw and preprocessed data.

For effective utilization of this dataset and code, we propose the following guidelines:

- Users can load the ‘processeddata’ files and apply the algorithms in ‘code.rar’ to decode this data. The results should align with those reported in our paper.
- Users can start by loading the ‘rawdata’ files and processing them using EEGLAB in MATLAB. Subsequently, the ‘code.zip’ algorithms can be used to decode this processed data, yielding results similar to those in our study.
- For further BCI research, users may load the ‘rawdata’ files and apply their own preprocessing and decoding codes.

## Supplementary information

Supplementary-data anonymization

## Data Availability

A script containing all the algorithms in this paper stored in ‘code.zip’ is provided with the dataset. All the code is implemented in Python 3.7.
