# Supplementary material for: A multi-day and high-quality EEG dataset for motor imagery brain-computer interface
Source: Sci Data. 2025 Mar 23;12:488. doi: 10.1038/s41597-025-04826-y (PMC11930978; doi:10.1038/s41597-025-04826-y)
Supplement: Supplementary file 1 — Supplementary-data anonymization [file 41597_2025_4826_MOESM1_ESM.docx]

**Data anonymization**

The dataset provided in this paper has undergone rigorous anonymization to protect the privacy of the volunteers. The main methods of anonymization used are as follows:

1. Volunteers only signed the survey questionnaire and informed consent form. We have removed personally identifiable information such as names, gender, age, phone numbers, and other sensitive details. The published data only includes the EEG data collected during the experimental process.
2. When publishing the data, all participants were named using identifiers (S001, S002, S003, ……). This ensures that the data cannot be directly linked back to an individual without additional information.
